# Research on Star Sensor Imaging Simulation Under Near-Space Hypersonic Non-Equilibrium Flow Conditions

**DOI:** 10.3390/s26030924

**Published:** 2026-01-31

**Authors:** Zhen Liao, Hongyuan Wang, Xi Cheng, Boqi Liu, Yunzhao Zang, Yinxi Lu, Shuai Yao, Zhiqiang Yan

**Affiliations:** Research Center of Space Optical Engineering, Harbin Institute of Technology, Harbin 150001, China; 20b921003@stu.hit.edu.cn (Z.L.); fountainhy@hit.edu.cn (H.W.); 120l011513@stu.hit.edu.cn (X.C.); l26698982669898@163.com (B.L.); 21b921002@stu.hit.edu.cn (Y.Z.); luyinxi1998@163.com (Y.L.); 23b921003@stu.hit.edu.cn (S.Y.)

**Keywords:** star sensors, hypersonic flow, aero-optical effects, imaging simulation

## Abstract

In order to address the difficulty in acquiring degraded images for star sensors under hypersonic conditions, this paper proposes a star sensor imaging simulation model which can effectively analyze the influence of thermochemical non-equilibrium flow on the star sensor. Firstly, the two-temperature model is adopted to describe the relaxation phenomenon of hypersonic non-equilibrium flow, and the chemical reaction is simulated by Arrhenius law. Then, the effects of optical transmission and thermal radiation on star sensor imaging are quantitatively analyzed. Based on this degradation model, the degraded star images under two typical working conditions are simulated. The simulation results show that the radiation of the solid media has the most significant influence on the imaging of the star sensor. The detectable limit magnitudes of the star sensor obtained under the two working conditions are 3.28 and 4.55, respectively. The research results can provide important theory and technical support for the system design and algorithm testing of star sensors on near-space hypersonic platforms.

## 1. Introduction

As a passive attitude measurement device without time accumulation error, star sensors are widely used in aerospace fields.

In recent years, near-space hypersonic vehicles have developed rapidly. This brings new application areas for star sensors. However, the aero-optics effect introduced by hypersonic vehicles will degrade the imaging quality of star sensors, which brings challenges to the near-space application of star sensors. At the same time, although concepts for hypersonic commercial aircraft have emerged, current hypersonic flight remains a specialized field and is not yet an accessible platform for routine observation. Due to the high cost of such vehicles, it is difficult to obtain real star maps through direct experiments [1]. Therefore, numerical simulation serves as a practical and economical means to obtain star images, supporting the theoretical design and algorithm testing of star sensors in the early development stage.

Near-space hypersonic vehicles typically fall into two categories: cruise vehicles operating at an altitude of around 30 km and glide vehicles flying at approximately 60 km. Both types significantly exceed the speed of conventional aircraft, presenting three key challenges for star image simulation. Firstly, aero-optical effects are divided into optical transmission effects and thermal radiation effects. The former refers to the change in the refractive index field that causes the wavefront of light to change, and the latter refers to the additional background radiation generated by the increase in temperature. Secondly, aero-optical effects usually involve two media, the flow field and the optical window, and the aero-optical effect mechanism produced by the two are different. Thirdly, the excessively high velocities also induce non-equilibrium flow phenomena, generally comprising chemical and thermal non-equilibrium, thereby introducing additional complexity to the star image simulation processes.

Early research regarding aero-optical effects in star sensor imaging primarily focused on optical transmission effects, utilizing Monte Carlo methods and Sutton’s model to compute the optical transfer function [2,3,4]. The flight speeds of the platforms involved in these studies are primarily in the range of Mach 3 to 6. Although speeds exceeding Mach 5 are generally classified as hypersonic, the flow conditions within this range are typically insufficient to induce significant thermal and chemical non-equilibrium phenomena. Thus, early studies did not pay enough attention to the influence of thermal radiation. Reference [5] analyzes the selection of star sensor observation windows for hypersonic platforms. The simulations account for thermal radiation effects under thermochemical non-equilibrium conditions. However, quantitative analysis of thermal radiation impacts on star sensor imaging is not included, limiting the comprehensiveness of the study. In terms of image simulation, multiple studies have addressed star image simulation; however, most prioritize atmospheric background radiation with limited attention to aero-optical effects [1,6]. References [7,8] investigate star sensor image simulation across a broad hypersonic velocity range (Mach 6–16). Although a relatively wide speed range has a certain degree of forward-looking, the study employs a simplified model that neither incorporates non-equilibrium phenomena in hypersonic flows nor accounts for radiation effects from solid media.

Consequently, this work achieves star image simulation under non-equilibrium flow conditions by implementing the two-temperature model to characterize hypersonic non-equilibrium relaxation phenomena and conducting quantitative analysis of aero-optical effects (both optical transmission and thermal radiation) on star tracker imaging. Section 2 introduces the physical models underpinning this research. Section 3 presents the quantitative analysis and evaluation methodology for aero-optical effects. Section 4 details the star image simulation process and results analysis under non-equilibrium flow conditions. Section 5 validates the CFD (Computational Fluid Dynamics) simulations and radiation computations implemented in this study. Section 6 is the conclusion of this paper.

## 2. Numerical Modeling of Non-Equilibrium Flow

### 2.1. Governing Equation

The conservation of mass, momentum, and energy in hypersonic non-equilibrium reacting flows is governed by the non-equilibrium Navier–Stokes–Fourier (NSF) equations. The flux-divergence form of this equation can be expressed as [9]

(1) $$\frac{\partial U}{\partial t}+\frac{\partial \left(F_{inv}-F_{vis}\right)}{\partial x_{i}}=W$$

The conserved quantities are defined as

(2) $$U={\left(\rho ,\rho_{s},\rho u_{1},\rho u_{2},\rho u_{3},E_{ve,m},E\right)}^{T}$$

where ρ and $\rho_{s}$ refer to the total density and partial density of component s, respectively. The index i represents the coordinate axes of the Cartesian coordinate system (i=1,2,3). $u_{i}$ is the velocity component. E and $E_{ve,m}$ are the total energy and vibro-electronic energy for molecule m. W is the source term. The inviscid flux $F_{i,inv}$ governs the convective transport of momentum and energy under the assumption of negligible molecular interactions, whereas the viscous flux $F_{i,vis}$ encapsulates diffusive transport mechanisms induced by intermolecular forces, including viscous stresses, heat conduction, and species diffusion. Their expressions are

(3) $$F_{i,inv}=\left(\begin{array}{l} \rho u_{i}\\ \rho_{s}u_{i}\\ \rho u_{i}u+\delta_{i1}p\\ \rho u_{i}v+\delta_{i2}p\\ \rho u_{i}w+\delta_{i3}p\\ E_{ve,m}u_{i}\\ \left(E+p\right) \end{array}\right),\;\;\;\;\;\;F_{i,vis}=\left(\begin{array}{l} 0\\ -J_{s,i}\\ \tau_{i1}\\ \tau_{i2}\\ \tau_{i3}\\ -q_{ve,i,m}-e_{ve,m}J_{m,i}\\ \tau_{ij}u^{j}-q_{tr,i}-q_{ve,i}-\sum_{r\neq e}h_{r}J_{r,i} \end{array}\right)$$

where $\delta_{ij}$ is Kronecker delta. p is the pressure. $J_{s,i}$ is the diffusive mass flux of species s (modeled via Fick’s law). $\tau_{ij}$ is the viscous stress tensor. $h_{r}J_{r,i}$ is the enthalpy transport due to species diffusion.

### 2.2. Non-Equilibrium Chemical Kinetic Model

Hypersonic speeds are usually referred to as speeds greater than Mach 5, but not all cases require consideration of chemical reaction models. At speeds below Mach 8, chemical reaction analysis is usually not required. When the speed of the hypersonic platform increases further, the outflow field will change into non-equilibrium flow. Non-equilibrium usually includes two aspects, namely chemical non-equilibrium and thermal non-equilibrium.

The most widely used model in this regard is Park’s two-temperature model, which assumes that dissociation and weak ionization reactions are controlled by the controlling temperature, expressed as

(4) $$T_{c}=T_{t}^{s}\cdot T_{v}^{s-1}$$

where T is the heavy particle translational and rotational temperature; $T_{v}$ is the vibrational and electron temperature. s is a weight factor that takes different values for different types of chemical reactions. The above two temperatures are used to describe the phenomenon that thermal equilibrium cannot be reached instantaneously due to the violent molecule collisions.

The forward Arrhenius law coefficient usually has the following expression:

(5) $$k_{f}=AT^{n}\exp(-T_{a}/T_{c})$$

where A is the pre-exponential factor, n is the temperature exponent, and $T_{a}$ is the temperature of activation. All of the forward coefficients used in this paper are shown in Table 1, M is the third body.

The reverse reaction rate is generally considered to be jointly determined by the forward reaction rate and the equilibrium constant. Park’s model postulates their relationship as follows:

(6) $$k_{r}=\frac{k_{f}(T_{c})}{k_{e}(T_{c})}$$

(7) $$k_{e}=\exp\left(a_{1}/\left(\frac{10000}{T_{c}}\right)+a_{2}+a_{3}\ln\left(\frac{10000}{T_{c}}\right)+a_{4}\left(\frac{10000}{T_{c}}\right)+a_{5}{\left(\frac{10000}{T_{c}}\right)}^{2}\right)$$

where coefficients $a_{1}$, $a_{2}$, $a_{3}$, $a_{4}$ and $a_{5}$ are determined by experimental fitting [12]. In addition, the effects of trace components in the air (H_2_O, OH, H, CO_2_, CO, C, NO_2_) on the reaction are also considered, and related reaction coefficient details can be found in reference [13].

### 2.3. Mesh and Boundary Condition

**Figure 1:**
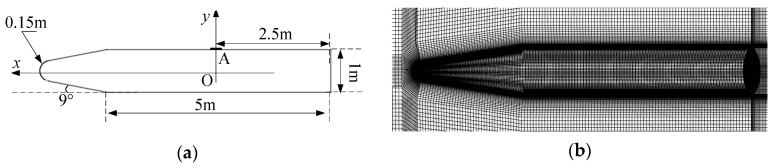
Hypothetical geometric model: (**a**) Hypothetical geometric model and (**b**) Structured grid.

In this paper, the hypothetical geometric model of the flight platform and the structured grid division results are shown in Figure 1. As illustrated in Figure 1a, a Cartesian coordinate system is established with the origin O (0,0) located on the symmetry axis directly below the optical window (point A), where the *x*-axis extends along the symmetry axis. The model has a total length of 7.348 m, and point A is the position of the optical window of the star sensor, which is located at the midpoint of the cylinder part.

**Figure 2:**
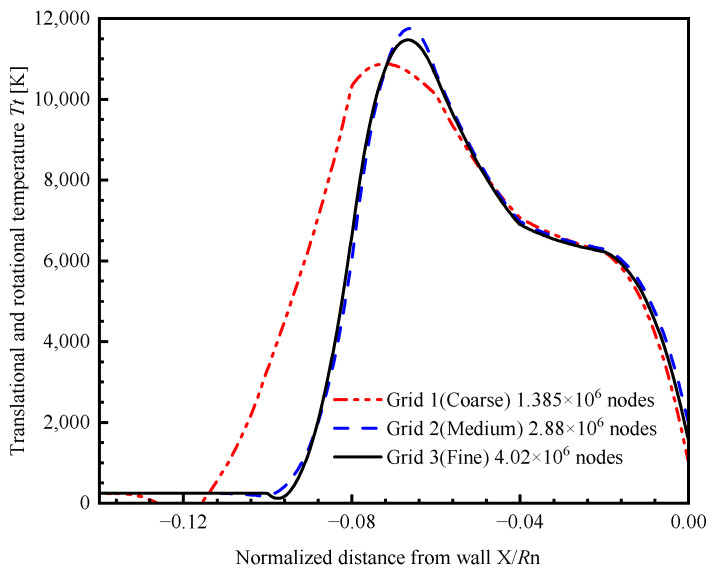
Translational and rotational temperature on stagnation stream-line.

To ensure the validity of subsequent computations, a grid independence study is required prior to the commencement of the simulation analysis. This paper chooses three sets of structured grids that share the same topological configuration but vary in the number of nodes to perform independence analysis. The flight height is 60 km with a speed of Mach 17.5. The calculation results of the $T_{t}$ on stagnation stream-line are shown in Figure 2. Considering the combined factors of computational accuracy and cost, Grid 2 is selected for subsequent analysis.

**Figure 3:**
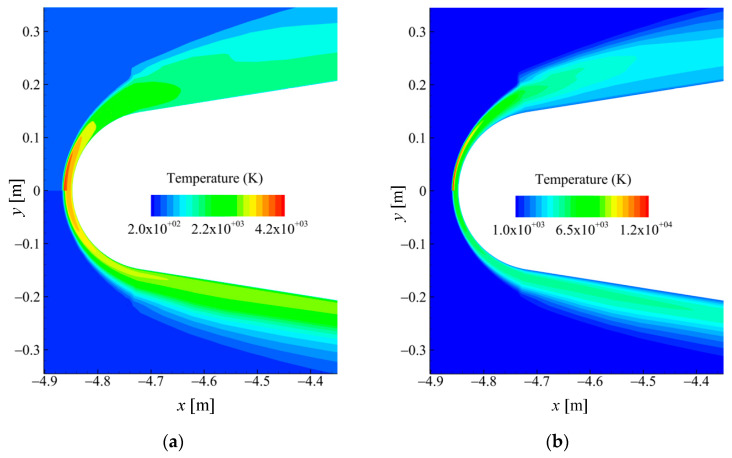
Spatial distribution of the simulated forebody temperature field: (**a**) 30 km 10 Ma (case 1) and (**b**) 60 km 17.5 Ma (case 2).

This paper considers two typical operating conditions of hypersonic vehicles. The first type is cruise vehicles, which typically fly at an altitude of around 30 km with a speed of approximately Mach 10. The second type is the glide vehicles, which are considered to fly at an altitude of 60 km and a speed of Mach 17.5. The specific boundary conditions are shown in Table 2:

Based on the geometric model and coordinate system defined in Figure 1, the contour plots of the simulated forebody temperature field are illustrated in Figure 3. For a clear comparison of the thermodynamic states, each sub-figure displays composite temperature fields separated by the symmetry axis (*y* = 0): the upper-half region (*y* > 0) represents the translational and rotational temperature $T_{t}$, while the lower-half region (*y* < 0) shows the vibrational and electron temperature ($T_{v}$). In case 1, the maximum temperature of $T_{t}$ is approximately 4300 K, and $T_{t}$ is relatively close to $T_{v}$, with the maximum temperature being about 3600 K. This indicates that the thermal non-equilibrium phenomenon is not prominent. In case 2, the maximum temperature of $T_{t}$ is approximately 12,500 K, while the maximum temperature of $T_{v}$ is merely around 6000 K, with a difference of nearly double. This suggests that the thermal non-equilibrium phenomenon in case 2 is significantly stronger than that in case 1.

**Figure 4:**
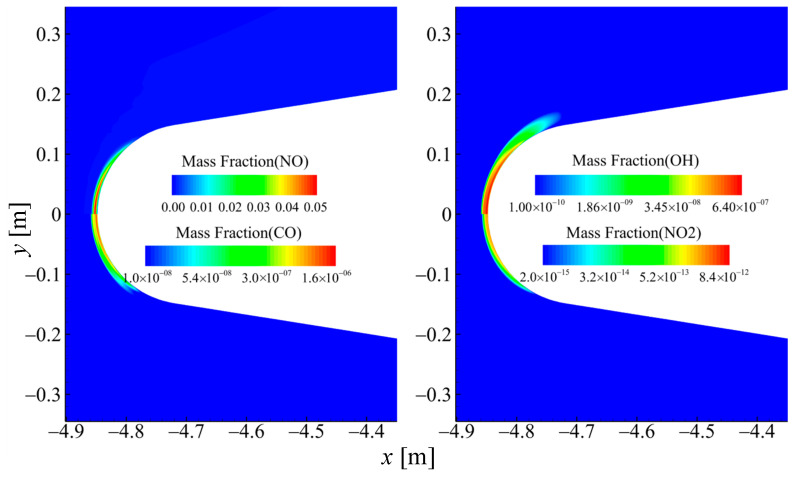
Spatial distributions of mass fractions for selected molecular species in case 2 (60 km 17.5 Ma).

**Figure 5:**
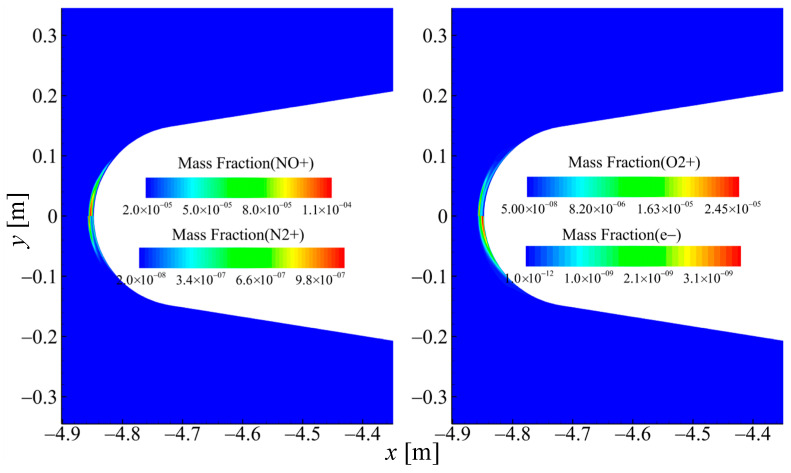
Spatial distributions of mass fractions for selected ionic species in case 2 (60 km 17.5 Ma).

Figure 4 presents the mass fraction contours of selected molecular species in case 2. The results show that the dissociated nitrogen and oxygen atoms produce a substantial amount of nitric oxide, with its mass fraction approaching 5% at the maximum. The molecules CO_2_ and H_2_O have undergone varying degrees of dissociation reactions near the wall surface, thus the mass fractions of CO and OH have increased significantly at the near-wall area. The mass fraction of NO_2_ is less than $1\times 10^{-10}$ at the maximum, and its concentration is so small that it can almost be ignored. Figure 5 presents the mass fraction contours of selected ionic species in case 2, among which NO^+^ exhibits a significantly higher concentration than the other species. The primary ionization region is localized at the model’s stagnation point.

### 2.4. Fluid Structure Interaction

For hypersonic vehicles, especially those with non-equilibrium flow at Mach 10 or above, the maximum temperature on their outer surfaces can even exceed 2000 K. Such high temperatures make it necessary to consider the thermal noise introduced by the heating of optical windows during star image simulation.

For the gas–solid interface, the following heat flux balance exists:

(8) $$q_{wn}=q_{c}-q_{rad}$$

where $q_{c}$ is the heat conduction into the structure; $q_{rad}$ is the radiative heat flux from the surface. $q_{wn}$ is the total heat flux normal to the wall, which can directly reflect the heat exchange effect of fluid on the wall surface, and its specific expression is

(9) $$q_{wn}=-\eta \frac{\partial T}{\partial n}-\eta_{v}\frac{\partial T_{v}}{\partial n}-\rho \sum h_{i}D_{i}\frac{\partial Y_{i}}{\partial n}$$

where η and $\eta_{v}$ are thermal conductivity and vibrational–electronic thermal conductivity respectively, $\frac{\partial T}{\partial n}$ is temperature gradient normal to the wall, ρ is the fluid density, i represents the a species, $h_{i}$ is the enthalpy, $D_{i}$ is the diffusion coefficient, and $\frac{\partial Y_{i}}{\partial n}$ is the mass fraction gradient normal to the wall.

$q_{c}$ is determined by the internal heat conduction of solids, and its expression is

(10) $$q_{c}=-\eta_{s}\nabla T_{s}\cdot \hat{n}$$

where $\eta_{s}$ is the structural thermal conductivity, $T_{s}$ is the structural temperature, and $\hat{n}$ is the surface unit normal vector.

**Figure 6:**
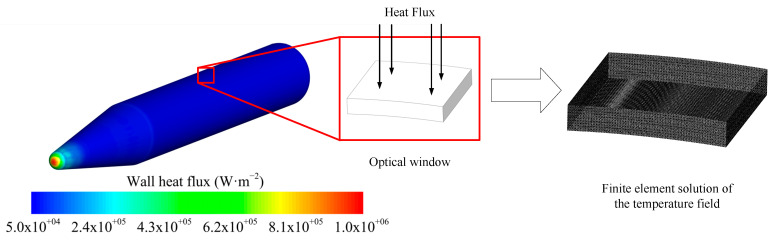
Schematic diagram of fluid structure interaction.

$q_{rad}$ can be calculated from the Stefan–Boltzmann Law:

(11) $$q_{rad}=\varepsilon \sigma T_{w}^{4}$$

where ε is the emissivity, σ is the Stefan–Boltzmann constant, and $T_{w}$ is the wall temperature. For the outer surface of aircraft, the current literature usually considers this value to be around 0.85. However, the optical window has a relatively low emissivity due to its high transmittance. In this paper, the optical window is modeled as diamond, and the emissivity is taken as 0.05 (sensitivity analysis presented in Section 4). The parameter information is shown in Table 3. The conformal optical window is 60 mm × 60 mm in size and 10 mm in thickness. Figure 6 shows the process of obtaining the optical window temperature through fluid structure interaction. Specifically, the spatially varying aerodynamic heat flux calculated by the CFD is extracted from the fluid–solid interface. This heat flux distribution is then mapped onto the external surface nodes of the optical window’s finite element model via interpolation, serving as the thermal boundary condition for the heat transfer analysis.

**Figure 7:**
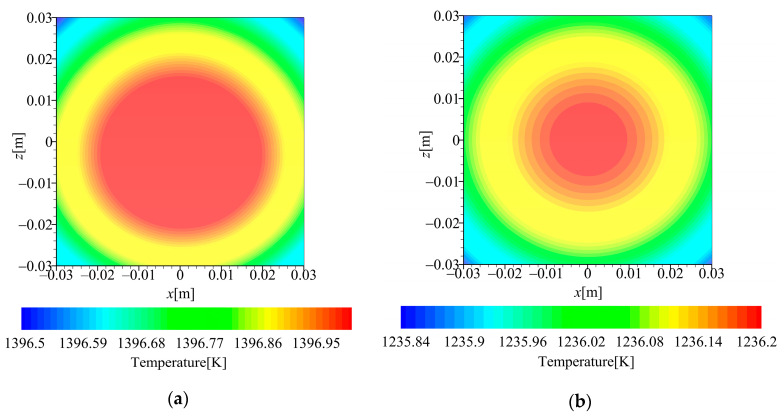
Simulation results of optical window temperature: (**a**) 30 km 10 Ma (case 1) and (**b**) 60 km 17.5 Ma (case 2).

The simulation results of the optical window temperature field obtained through fluid structure interaction are shown in Figure 7. Although case 2 has a higher speed, the significantly lower atmospheric density at 60 km rather than 30 km leads to reduced aerodynamic heating. Consequently, the optical window temperature is somewhat lower than in case 1.

## 3. Aero-Optical Effects Analysis

### 3.1. The Optical Transmission Effects

The phenomenon that the flight process of the vehicle causes changes in the density distribution of the flow field and thereby alters the transmission of light is called the optical transmission effect.

The optical transmission effect can be divided into two parts: laminar flow and turbulent flow. Regarding the laminar flow component, assuming that the light is incident perpendicularly, the OPD (optical path difference) at the entrance pupil can be obtained through ray tracing, and then the generalized pupil function A(x,y) expression can be written as

(12) $$A(x,y)=a(x,y)\exp[j\frac{2\pi }{\lambda }w(x,y)]$$

where a(x,y) is the pupil function; w(x,y) is the wave aberration. Based on Huygens’s principle, the amplitude distribution in the image plane $U(x^{\prime },y^{\prime })$ is as follows:

(13) $$U(x^{\prime },y^{\prime })=\iint A(x,y)\exp[-j\frac{2\pi }{\lambda f^{\prime }}(xx^{\prime }+yy^{\prime })]dxdy$$

Since the light intensity is the square of the amplitude, the point spread function of the laminar flow components can be written as [2]

(14) $$PSF_{\mathrm{laminar}}(x^{\prime },y^{\prime })={\left|U(x^{\prime },y^{\prime })\right|}^{2}$$

Calculating the OPD is a key step in analyzing the optical transmission effect. The optical path is defined as the product of the geometric path length that light travels and the refractive index of the medium, where the refractive index $n_{\mathrm{Refraction}}$ is conventionally characterized by the Gladstone–Dale relation:

(15) $$n_{\mathrm{Refraction}}=K_{\mathrm{GD}}\rho +1$$

(16) $$K_{\mathrm{GD}}=2.23\times 10^{-4}\left(1+\frac{7.52\times 10^{-3}}{{\left(\lambda \times 10^{6}\right)}^{2}}\right)$$

where $K_{\mathrm{GD}}$ is the Gladstone–Dale coefficient, ρ is the density of air, and λ is the optical wavelength. It is evident that the refractive index field is predominantly governed by the density field.

Usually, viscous heating on the vehicle’s external surface induces a reduction in flow field density. Some previous studies have analyzed the conditions at an altitude of approximately 20 km. In such conditions, the atmosphere still has considerable density, and thus the transmission effect is obvious [2,17].

**Figure 8:**
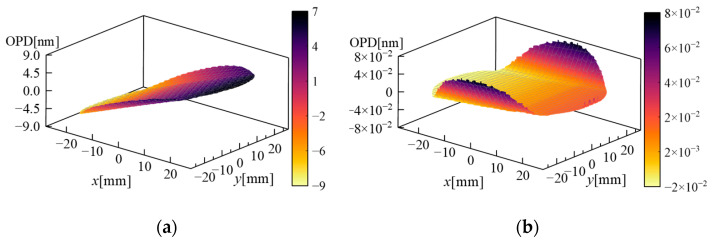
The OPD simulation results: (**a**) Case 1 and (**b**) Case 2.

However, the flight altitude in this paper is higher, and the atmosphere is thinner. This condition reduces the impact of transmission effects on imaging. Figure 8 presents the simulated optical path difference (OPD) results. According to the Rayleigh criterion, the impact on star sensor imaging is negligible when the OPD is less than 1/4 λ. Therefore, the effect of laminar flow components on star sensor imaging is negligible.

The exposure time of the star sensor is typically in the millisecond range. Under hypersonic conditions, according to reference [18], the star sensor system can be regarded as a long-exposure system. For the turbulent flow component, The SR (Strehl ratio) is usually used to describe its attenuation of light intensity. Its expression is as follows:

(17) $$SR_{\mathrm{turbulent}}=\exp\left[-\left(\frac{2\pi {\mathrm{OPD}}_{\mathrm{rms}}}{\lambda }\right)\right]$$

where ${\mathrm{OPD}}_{\mathrm{rms}}$ is the root mean square of the OPD. Based on the simulation results of OPD, the $SR_{\mathrm{turbulent}}$ can be calculated as 0.965 of case 1, this indicates that turbulence has caused the starlight to lose approximately 3.5% of its energy. For case 2, the extremely thin atmosphere results in a $SR_{\mathrm{turbulent}}$ almost equal to 1, suggesting that the attenuation of starlight by turbulence is almost negligible.

### 3.2. The Thermal Radiation Effects

The radiative transfer equation can be written as

(18) $$\frac{dL}{ds}=-I_{A}L+I_{E}$$

where L is the radiance, $I_{A}$ is the absorption coefficient, $I_{E}$ is the emission coefficient, and s is the transmission path. After organizing this equation and discretizing it according to the propagation direction, we have

(19) $$L_{n+1}=L_{n}\cdot \exp\left(-I_{A}\Delta s\right)+\frac{I_{E}}{I_{A}}\left(1-\exp(-I_{A}\Delta s)\right)$$

where $L_{n}$ is the radiance of the nth layer medium. This formula is also called the LOS (light of sight) method.

For solid media, the total radiance includes both its self-emission and the transmitted incident radiance. Assuming negligible reflection, the total radiance $L_{total}$ entering the sensor is expressed as

(20) $$L_{total}=\varepsilon B(T_{w})+(1-\varepsilon )L_{n}$$

where ε is the emissivity, $T_{w}$ is the temperature of the solid medium, $B(T_{w})$ is the Planck blackbody radiation, and $L_{n}$ is the incident radiance from the adjacent gas layer.

For fluid media, the process of solving the absorption and emission coefficients is relatively complex. At low flight speeds, only radiation from H_2_O and CO_2_ typically needs to be considered. However, as the speed increases further, a substantial amount of NO gas generated by chemical reactions also becomes a significant radiative source. Additionally, due to the thermal non-equilibrium state of the gas, radiation calculations require the use of multi-temperature models. Given the limited space, further implementation details can be found in reference [19].

**Figure 9:**
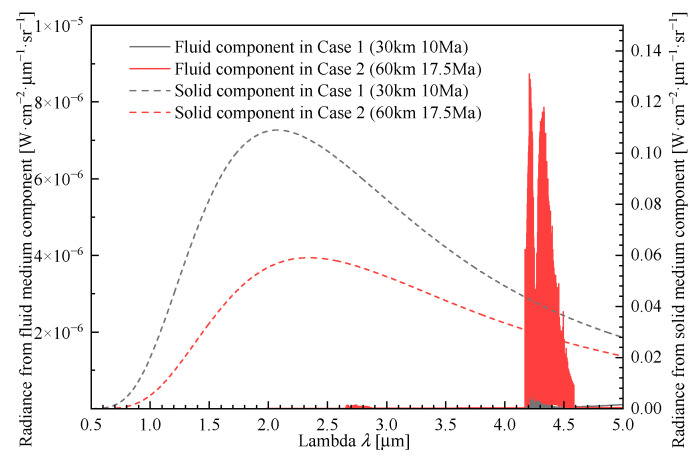
Radiation simulation results.

Based on the above model, the radiation simulation results can be obtained as shown in Figure 9. It can be observed that under both case 1 and case 2 operating conditions, the radiation generated by the solid medium dominates and exceeds that of the fluid medium by several orders of magnitude. The fluid medium exhibits distinct characteristic peaks at 2.7 μm and 4.3 μm, primarily attributed to the CO_2_ *v*_1_ + *v*_3_ combination band and the CO_2_ *v*_3_ fundamental vibrational band (similar computational results are also reported in many references [20]). Current star sensor systems predominantly utilize detectors in the visual band or SWIR (short-wave infrared) band. To enhance visualization, the computational range is extended from the visual to MWIR (mid-wave infrared) spectrum; however, subsequent analyses will focus solely on the visual and SWIR radiation results.

## 4. Star Map Simulation

### 4.1. Star Map Simulation Process

**Figure 10:**
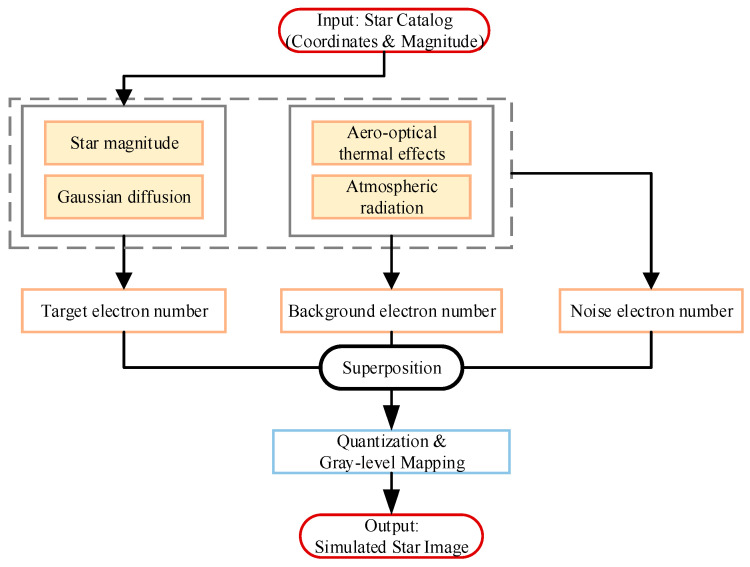
Star image simulation flowchart.

The comprehensive star image simulation framework established in this study is illustrated in Figure 10. The final output of this simulation is the digital gray level, denoted as G, which is determined by the quantization of the total accumulated electrons $S_{\mathrm{total}}$ within the exposure time. The specific mathematical model is expressed as

(21) $$G=\left\lfloor \frac{S_{\mathrm{total}}}{N_{\mathrm{full}}}\left(2^{b}-1\right)+0.5\right\rfloor$$

where $S_{\mathrm{total}}$ is the electron number, $N_{\mathrm{full}}$ is the full well depth, and b is the digital output format. $\left\lfloor \cdot \right\rfloor$ is the floor function to ensure integer outputs. As indicated in the signal flow of Figure 10, the total electron number $S_{\mathrm{total}}$ is mathematically modeled as the superposition of three distinct components:

(22) $$S_{\mathrm{total}}=S_{\mathrm{target}}+S_{\mathrm{bg}}+S_{\mathrm{noise}}$$

where $S_{\mathrm{target}}$ represents the electrons generated by the target star. $S_{\mathrm{bg}}$ denotes the electrons from the background stray light. $S_{\mathrm{noise}}$ accounts for the system noise. In the simulation, $S_{\mathrm{noise}}$ is primarily composed of shot noise and detector dark current. The shot noise adheres to Poisson statistics, with its standard deviation dependent on the incident signal strength ($S_{\mathrm{target}}+S_{\mathrm{bg}}$), while dark current is incorporated based on the specific detector parameters.

#### 4.1.1. Target Electron Number

The number of electrons generated by the target star, denoted as $S_{m}$, is calculated based on the target magnitude and optical system parameters. The equation is formulated as

(23) $$S_{m}=\frac{\pi D^{2}\cdot \tau_{\mathrm{opt}}\cdot t_{\mathrm{int}}\cdot E_{m}\cdot Q_{e}}{4}\int_{\lambda_{2}}^{\lambda_{1}}\frac{1}{W_{\mathrm{ph}}}d\lambda$$

where *m* is the star magnitude; $S_{m}$ is the number of electrons generated by the target on the image plane when the magnitude is *m*. $\tau_{\mathrm{opt}}$ is the transmittance of optical system. $t_{\mathrm{int}}$ is the exposure time. *D* is the entrance pupil diameter. $W_{\mathrm{ph}}$ is the single-photon energy. $Q_{e}$ is the quantum efficiency. The variable $E_{m}$ represents the in-band irradiance of the target, which is quantitatively determined by the stellar magnitude *m*. Following the fundamental definition of the astronomical magnitude system, this relationship is expressed as

(24) $$E_{m}(\lambda )=E_{0}\times 2.512^{-m}$$

where $E_{0}$ denotes the reference irradiance of a zero-magnitude star (*m* = 0). It is important to note that $E_{0}$ is not a universal constant but depends on the specific photometric system and the spectral band used. Previous research suggests that in near-space environments, employing the short-wave infrared (SWIR) band instead of the visual band can effectively mitigate atmospheric background radiation [21]. To investigate the optimal spectral band selection for star sensors in near-space environments, this study conducts simulations covering both the visual and SWIR bands. Accordingly, the adopted photometric systems and their corresponding zero-magnitude reference parameters are listed in Table 4. It should be noted that $E_{0}$ is derived as the product of the zero-magnitude spectral irradiance and the effective bandwidth.

#### 4.1.2. Background Electron Number

The background electron number, denoted as $S_{\mathrm{bg}}$, represents the noise signal generated by stray light entering the optical system. In the near-space hypersonic scenario, the total background radiance $L_{\mathrm{bg}}(\lambda )$ primarily originates from two sources: the aero-optical thermal radiation (comprising emissions from both the non-equilibrium flow field and the heated optical window) and the atmospheric background radiation. By integrating this radiance over the solid angle of the instant field of view (IFOV), the equation is formulated as

(25) $$S_{\mathrm{bg}}=\frac{\pi D^{2}\cdot \tau_{\mathrm{opt}}\cdot t_{\mathrm{int}}\cdot \Omega \cdot Q_{e}}{4}\int_{\lambda_{2}}^{\lambda_{1}}\frac{L_{\mathrm{bg}}(\lambda )}{W_{\mathrm{ph}}}d\lambda$$

where Ω is the solid angle of single pixel.

**Figure 11:**
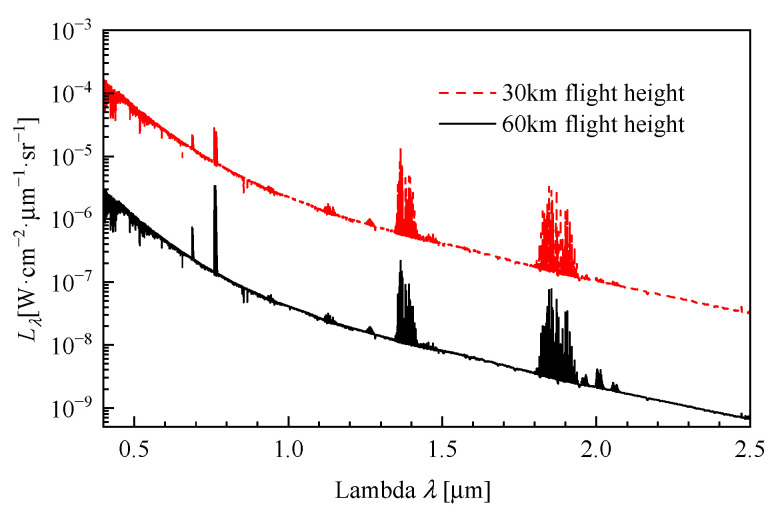
Atmospheric background radiation calculation results by MODTRAN.

In the calculation of $S_{\mathrm{bg}}$, the component of $L_{\mathrm{bg}}(\lambda )$ arising from aero-optical thermal radiation adopts the analysis results from Section 3. For the remaining component—the atmospheric background radiation—quantitative data is acquired through simulation. In this study, we utilize MODTRAN (Moderate Resolution Atmospheric Transmittance and Radiance Code) code to generate the atmospheric spectral radiance [6,24]. The simulation results (solar zenith angle = 0°, angle between line of sight and solar azimuth = 0°) are presented in Figure 11.

### 4.2. System Parameter Setting

For the convenience of discussion, this paper selects a detector that can simultaneously cover the visual and SWIR bands, and realizes the imaging simulation of different spectral bands through the form of a filter (the spectral response curve in the visual band is aligned with the VT band of the Hipparcos catalog, while the spectral response curve in the SWIR band matches the H-band of the 2MASS (Two Micron All-Sky Survey) catalog). The specific parameters of the star sensor system are shown in Table 5.

### 4.3. Analysis of Star Image Simulation Results

**Figure 12:**
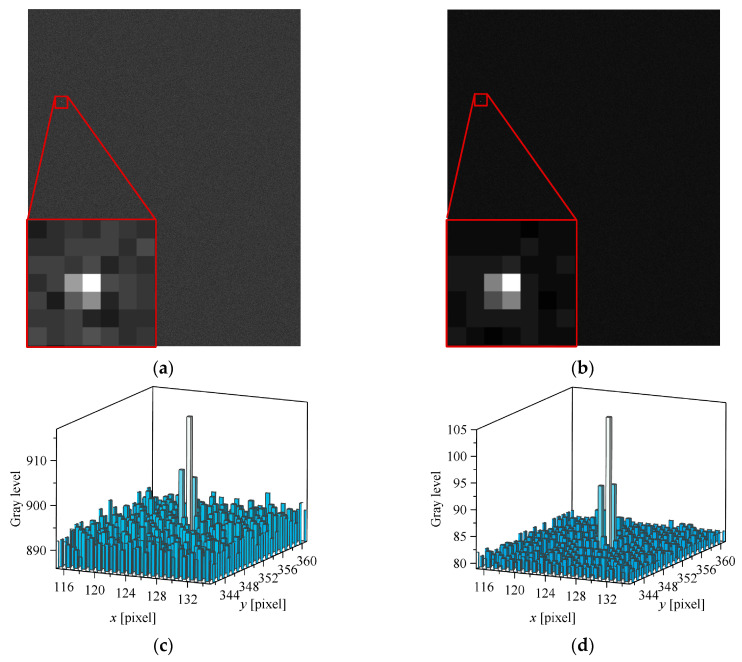
Star image in visual band simulation results: (**a**) Case 1 simulation star image; (**b**) Case 2 simulation star image; (**c**) Case 1 star point local gray level distribution and (**d**) Case 2 star point local gray level distribution. (The red frames indicate the region of interest for local magnification, and the 3D columns represent the local gray-level distribution of the star point.)

Figure 12 shows simulated star images in the visual band for case 1 and case 2. In Figure 12a, the red-outlined region at the lower-left corner provides a magnified view of the brightest star (Hipparcos ID = 112,029, RA = 340.36°, Dec = 10.83°, Magnitude in Hipparcos VT band = 3.40) within the simulation star image. Figure 12c displays the localized grays level distribution of this star. Notably, although the star is relatively bright, the background gray level remains comparable to the peak gray level of the star point. Significant fluctuations in the background gray level (noise) are evident. This demonstrates that aero-optical thermal radiation effects critically degrade the imaging process of the star sensors. In comparison, case 2 exhibits significantly lower noise levels in its simulated star images due to reduced aero-optical thermal radiation effects compared to case 1, resulting in superior imaging quality.

**Figure 13:**
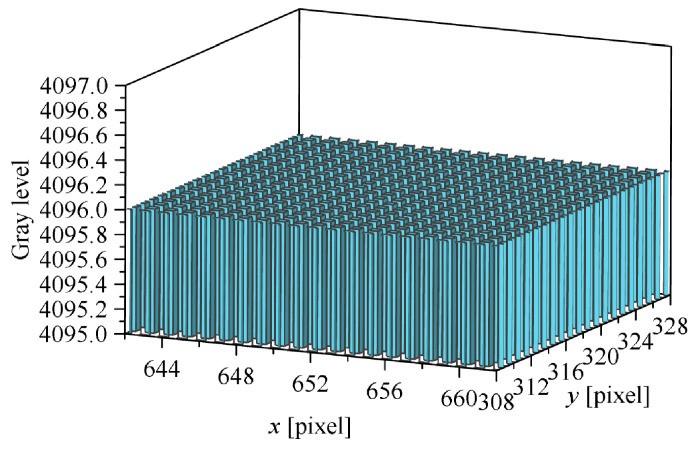
Star image in SWIR band simulation results.

Under hypersonic non-equilibrium flow conditions, due to the significant increase in aerothermal radiation, the idea of using star sensors in the SWIR band to reduce the atmospheric background radiation is no longer feasible. Figure 13 presents the simulated star image results for case 2 in the SWIR band (other parameters remain unchanged from previous settings). The entire focal plane exhibits saturation due to background thermal radiation being dominated by emission from solid media rather than atmospheric background radiation. As the spectral peak of this radiation follows Wien’s displacement law, reducing the wavelength helps mitigate background radiation effects. Thus, for flight platforms equipped with star sensors operating at extreme velocities where the external flow field is in thermochemical non-equilibrium, the use of Vt band star sensors is strongly recommended.

To further elucidate the impact of thermal radiation on star sensor imaging degradation, SNR (signal-to-noise ratio) analysis is employed to quantify detection capability. The expression of SNR can be written as

(26) $$\mathrm{SNR}=\frac{K_{0}S_{m}}{\sqrt{K_{0}S_{m}+S_{bgk}+I_{dark}\cdot t_{int}+\sigma_{read}^{2}}}$$

where $K_{0}$ is the ratio of the secondary central pixel energy of the star point to the total energy, and for a 3 × 3 pixel star point, the value is 0.1136.

**Figure 14:**
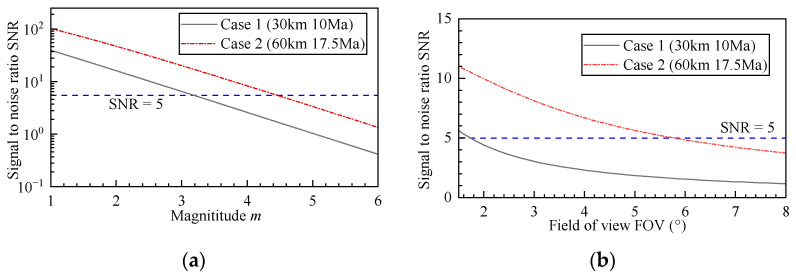
Variation of signal-to-noise ratio with star point: (**a**) The relationship between magnitude and SNR and (**b**) The relationship between field of view and SNR.

The simulation results of different magnitudes of SNR of case 1 and case 2 are shown in Figure 14a. As the star magnitude increases (indicating reduced target irradiance), the star point SNR decreases monotonically. A SNR > 5 is typically considered the detection threshold for star points [25,26]. Thus, the detectable limit magnitude for case 1 and case 2 are obtained as 3.28 and 4.55, respectively. Below this threshold, the secondary peak of the stellar point merges with background noise, making it difficult to segment the star’s connected domain. It is evident that thermal radiation significantly compromises the detection limit capability of the star sensor. Consequently, in subsequent designs of such systems, increasing the focal length and reducing the field of view (thereby decreasing the solid angle of single pixel) should be implemented to enhance detection performance.

To further guide the optical system design, an analysis of the maximum field of view is conducted. Taking a detectable limit magnitude of 5 as an example, the simulation results for the signal-to-noise ratio variation under different field of view conditions (achieved by varying the focal length while keeping other parameters constant) are shown in Figure 14b. As the field of view increases, the signal-to-noise ratio gradually decreases. Therefore, it can be concluded that under case 1 and case 2, the field of view of the star sensor should be less than 1.8° and 5.8°, respectively.

Given that the emissivity of 0.05 is a relatively extreme choice, a sensitivity analysis is conducted to assess its impact. Table 6 illustrates the changes in the detectable limit magnitude for case 1 and case 2 when the emissivity varies from 0.05 to 0.25.

**Figure 15:**
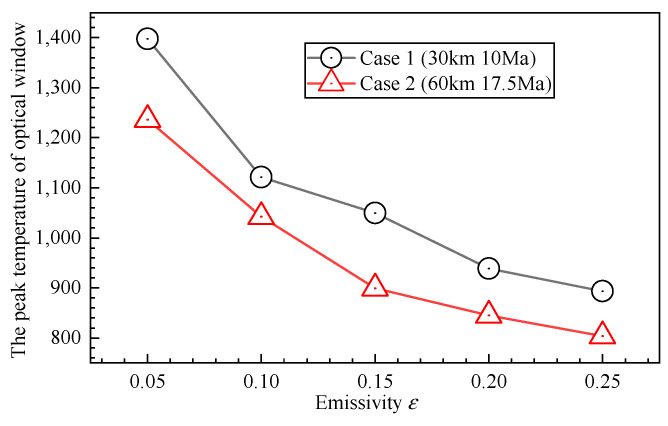
The relationship between the peak temperature of the optical window and the emissivity.

It can be observed that the detectable limit magnitude increases as the emissivity increases. This indicates that a higher emissivity mitigates the impact of aerodynamic thermal radiation on the star sensor imaging. This is because under a fixed external aero-heating condition, increasing the emissivity enhances radiative cooling, resulting in a lower steady-state window temperature. Figure 15 shows the relationship between the peak temperature of the optical window and the emissivity. Although emissivity enters the radiation term linearly, thermal radiation depends on the fourth power of temperature; therefore, the reduction in temperature dominates, leading to a decrease in radiative intensity within the Vt band. Moreover, according to Wien’s displacement law, the decrease in temperature shifts the radiation peak toward longer wavelengths, further reducing the contribution in the visible spectrum. However, as the emissivity continues to rise, the thermal radiation from the optical window eventually decreases to a level comparable to the atmospheric background, ceasing to be the dominant noise source. At this stage, the negative impact of high emissivity becomes apparent: an increase in emissivity leads to a corresponding decrease in transmissivity. This attenuates the incoming starlight signal and reduces the signal-to-noise ratio (SNR), causing the detectable limit magnitude to decline after reaching a peak.

## 5. Validation

Research related to fluid mechanics often encounters challenges in conducting direct validation experiments due to the high costs involved. Therefore, this paper will employ a comparative approach, juxtaposing simulation results with classical case studies and published experimental data to validate the accuracy of the simulations presented herein.

### 5.1. Validation Case 1

**Figure 16:**
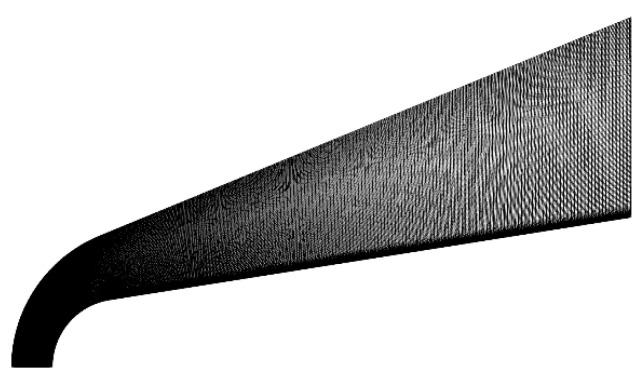
RAM C-II meshing results.

The RAM C-II (Radio Attenuation Measurement C-II) experiment, conducted by NASA in 1970, aimed to investigate plasma sheath properties during high-speed atmospheric reentry. In this validation case, this paper reproduces the experimental conditions by simulation means, and compares the simulation results with others’ simulation or experimental data to illustrate the correctness of the CFD simulation results in this paper. The RAM C-II model has a total length of 1.3 m, a ball head radius of 1.524 m, a cone angle of 9°, and the meshing results are shown in Figure 16. The number of grid nodes is 200 × 180, and the height of the first layer is set to 5 × 10^−5^ m. The reproduced working conditions are shown in Table 7.

**Figure 17:**
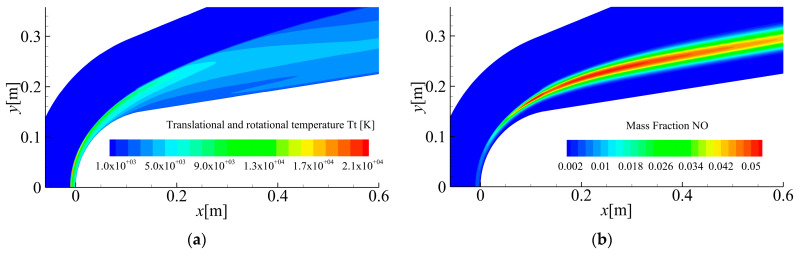
Translational and rotational temperature and mass fraction of NO simulation results with Mach 23.9: (**a**) Translational and rotational temperature and (**b**) Mass fraction of NO.

Figure 17 illustrates the simulated translational–rotational temperatures and NO mass fractions at Mach 23.9, showing peak values exceeding 20,000 K and 5%, respectively. The extremely high temperature causes the gas to produce a significant ionization effect, producing a large number of free electrons.

Table 8 presents a comparison with other simulation results under the condition of Mach 23.9. It is evident that the simulation results in this study fall within a reasonable range for all parameters, including detachment distance, peak temperature, and NO^+^ mass fraction, and are largely consistent with those reported in other studies.

**Figure 18:**
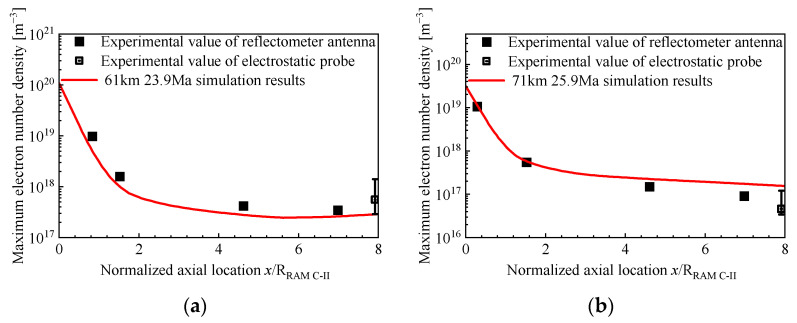
Maximum electron number densities along the body for RAM C-II: (**a**) 23.9 Ma and (**b**) 25.9 Ma.

The electron number density directly reflects the degree of ionization of the non-equilibrium flow. Figure 18 shows the comparison between the maximum axial electron number of RAM C-II in this paper and the experimental data [30]. The simulation results are in good agreement with the experimental data, which further demonstrates that the non-equilibrium simulation results in this paper are reliable.

### 5.2. Validation Case 2

**Figure 19:**
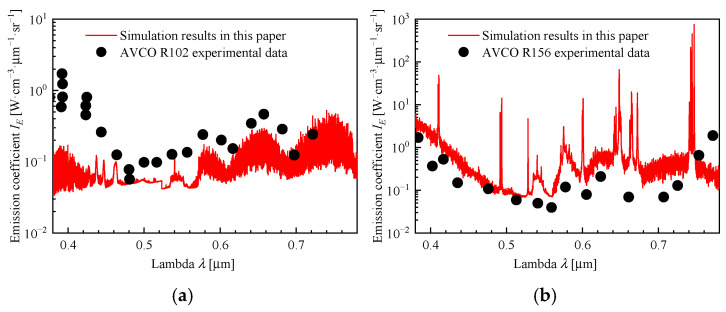
The comparison between the simulation results and the shock tube experiments by AVCO Everett Lab: (**a**) Equilibrium R102 and (**b**) Non-equilibrium R156.

The R102 and R156 experiment data, derived from shock-tube studies conducted by AVCO Everett Laboratories, encompass equilibrium nitrogen (R102) and non-equilibrium air (R156) under hypersonic conditions [31,32]. These datasets are employed in this study to validate the radiation code’s predictive capability across both equilibrium and multi-temperature non-equilibrium regimes. Table 9 presents the fluid data of the experiment, and the radiation calculation results of the fluid are shown in Figure 19.

### 5.3. Validation Case 3

NASA TP-2334 provides an extensive experimental database of Mach 10 surface heat-flux distributions for a biconic configuration [33]. The lower-surface heat-flux data obtained at a 10° angle of attack is adopted in the present work for validation.

**Figure 20:**
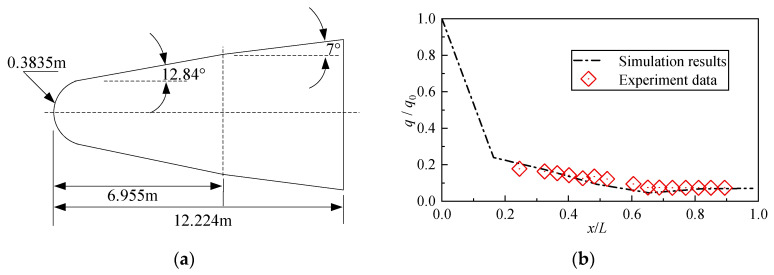
The comparison between the simulation results and the experimental data of NASA TP-2334: (**a**) Geometric dimensions and (**b**) Comparison results.

Figure 20a shows the geometric dimensions of the model, with a total length of L=12.224 m, and Figure 20b presents the comparison between the simulation results and the experimental data. It can be observed that the simulation results exhibit the same variation trends as the experimental measurements and show good overall agreement. This consistency demonstrates that the numerical approach adopted in the present study is reliable and adequate for simulating heat transfer under the present conditions.

## 6. Conclusions

The main conclusions of this paper include the following two points:

(1) This study establishes an imaging degradation model for star sensors under near-space hypersonic non-equilibrium flow conditions. The model quantitatively evaluates the impacts of optical transmission effects and thermal radiation on star sensor imaging, revealing that thermal radiation from solid media is the main factor affecting the imaging of star sensors.
(2) A simulation framework for star sensor imaging under near-space hypersonic non-equilibrium flows is developed. The framework performs star image simulations for two typical hypersonic platform operational scenarios, generating degraded star images. It obtains detectable limit magnitudes of 3.28 and 4.55 for case 1 and case 2, respectively.

## Figures and Tables

**Table 1:** Chemical reactions and rate coefficients [10,11].

Number	Reaction	Reaction Type	*A* (cm^3^·mol^−1^·s^−1^)	*n*	*T_a_* (K)
1	$O_{2}+M⇌2O+M$	Dissociation	2 × 10^21^	−1.50	59,500
2	$N_{2}+M⇌2N+M$	Dissociation	7 × 10^18^	−1.60	113,200
2	NO+M⇌N+O+M	Dissociation	5 × 10^12^	0.00	75,500
4	$N_{2}{+e}^{-}⇌{2N+e}^{-}$	Dissociation	1.2 × 10^25^	−1.60	113,200
5	$\mathrm{NO}+O⇌O_{2}+N$	NO Exchange	8 × 10^12^	0.00	19,450
6	$N_{2}+O⇌\mathrm{NO}+N$	NO Exchange	6.4 × 10^17^	−1.00	38,400
7	$N+O⇌{\mathrm{NO}}^{+}+e^{-}$	Associative Ionization	5.3 × 10^12^	0.00	31,900
8	$N+N⇌N_{2}^{+}+e^{-}$	Associative Ionization	4.4 × 10^7^	1.50	67,500
9	$O+O⇌O_{2}^{+}+e^{-}$	Associative Ionization	7.1 × 10^2^	2.70	80,600
10	${\mathrm{NO}}^{+}+O⇌N^{+}+O_{2}$	Charge Exchange	1.0 × 10^12^	0.50	77,200
11	$N^{+}+N_{2}⇌N_{2}^{+}+N$	Charge Exchange	1.0 × 10^12^	0.50	12,200
12	$O_{2}^{+}+N⇌N^{+}+O_{2}$	Charge Exchange	8.7 × 10^13^	0.14	28,600
13	$O^{+}+\mathrm{NO}⇌N^{+}+O_{2}$	Charge Exchange	1.4 × 10^5^	1.90	26,600
14	$O_{2}^{+}+N_{2}⇌N_{2}^{+}+O_{2}$	Charge Exchange	9.9 × 10^12^	0.00	40,700
15	$O_{2}^{+}+O⇌O^{+}+O_{2}$	Charge Exchange	4.0 × 10^12^	−0.09	18,000
16	${\mathrm{NO}}^{+}+N⇌O^{+}+N_{2}$	Charge Exchange	3.4 × 10^13^	−1.08	12,800
17	${\mathrm{NO}}^{+}+O_{2}⇌O_{2}^{+}+\mathrm{NO}$	Charge Exchange	2.4 × 10^13^	0.41	32,600
18	${\mathrm{NO}}^{+}+O⇌O_{2}^{+}+N$	Charge Exchange	7.2 × 10^12^	0.29	48,600
19	$O^{+}+N_{2}⇌N_{2}^{+}+O$	Charge Exchange	9.1 × 10^11^	0.36	22,800
20	${\mathrm{NO}}^{+}+N⇌N_{2}^{+}+O$	Charge Exchange	7.2 × 10^13^	0.00	35,500
21	$O+e^{-}⇌O^{+}+2e^{-}$	Electron-Impact Ionization	3.9 × 10^33^	−3.78	158,500
22	$N+e^{-}⇌N^{+}+2e^{-}$	Electron-Impact Ionization	2.5 × 10^34^	−3.82	168,600

**Table 2:** The boundary conditions of flow field simulation.

	Flight Altitude (km)	Pressure (Pa)	Temperature (K)	Velocity (Mach)
Case 1	30	1197	226.51	10.0
Case 2	60	21.96	247.02	17.5

**Table 3:** Physical constants and material properties used in the aero-optical thermal simulation.

Category	Parameters	Value	Unit	References
Constants	Stefan–Boltzmann constant	5.67 × 10^−8^	W·m^−2^·K^−4^	-
	Planck constant	6.626 × 10^−34^	J·s	-
	Boltzmann constant	1.380 × 10^−23^	J·K^−1^	-
Material (Diamond)	Emissivity	0.05	-	[14]
	Thermal Conductivity	500 (at 1000 K)	W·m^−1^·K^−1^	[15]
Material (Fuselage)	Wall Emissivity	0.85	-	[16]

**Table 4:** Zero-magnitude attributes of the star catalog [22,23].

Star Catalog	Band Filter	Lambda (μm)	Bandwidth (μm)	Spectral Irradiance (W·cm^−2^·μm^−1^)
Tycho2/Hipparcos	Vt	0.535	0.167	4.029 × 10^−12^
2MASS	H	1.662	0.251	1.133 × 10^−13^

**Table 5:** System simulation parameters of star sensor.

Parameter		Value	Unit
Optical system	Focal length	156	mm
	Entrance pupil diameter	55.7	mm
	F-number	2.8	-
	Transmittance of optical system	85	%
Sensor	Active pixel	1280 × 1024	−
	Pixel pitch	15 × 15	μm
	Spectral response	0.4–1.7	μm
	Digital output format	12	bit
	Full well capacity	1,800,000	e^−^
	Exposure time	10	ms
	Quantum efficiency	0.7	%
	Noise with ROIC	<40	e^−^
	Dark current	187,245	(e^−^)/pixel/s

**Table 6:** The detectable limit magnitudes under different emissivity conditions.

	Detectable Limit Magnitude with Different Emissivities				
	0.05	0.10	0.15	0.20	0.25
Case 1	3.28	4.22	4.23	4.19	4.12
Case 2	4.55	5.55	5.73	5.67	5.61

**Table 7:** Boundary condition of the RAM C-II.

	Free Stream Velocity (Ma)	Flight Altitude (km)	Pressure (Pa)	Temperature (K)
Condition 1	23.9	61	19.7	244.0
Condition 2	25.9	71	4.9	219.6

**Table 8:** Simulation results along the stagnation line for RAM C-II with Mach 23.9.

Results	Ghislain [27]	Candler [28]	Josyula [29]	This Paper
Normalized detachment distance (%)	6.75	9.18	7.21	7.22
$\mathrm{Peak}\;\mathrm{of}\;T_{t}$ (K)	18,400	22,500	20,500	20,528
Peak of mass fraction NO^+^ (%)	0.313	0.164	0.1	0.219

**Table 9:** The fluid data of the R102 and R156.

Parameters	Unit	AVCO R102	AVCO R156
Mole Fractions of N	%	1.878 × 10^−1^	4.813 × 10^−1^
Mole Fractions of N^+^	%	1.800 × 10^−5^	3.425 × 10^−2^
Mole Fractions of O	%	–	1.788 × 10^−1^
Mole Fractions of O^+^	%	–	6.204 × 10^−1^
Mole Fractions of N_2_^+^	%	1.500 × 10^−5^	6.188 × 10^−4^
Mole Fractions of N_2_	%	8.121 × 10^−1^	2.143 × 10^−1^
Mole Fractions of NO	%	–	3.032 × 10^−2^
Mole Fractions of O_2_	%	–	1.160 × 10^−2^
Mole Fractions of e^−^	%	3.300 × 10^−5^	4.269 × 10^−2^
Total Number Density	cm^−3^	3.604 × 10^18^	8.013 × 10^16^
Translational and Rotational Temperature	K	6279	18,000
Vibrational Temperature	K	6279	14,000
Electronic Temperature	K	6279	12,000

## Data Availability

The original contributions presented in this study are included in the article. Further inquiries can be directed to the corresponding author.
